# Enhanced Isolation of *Streptomyces* from Different Soil Habitats in Calamba City, Laguna, Philippines using a Modified Integrated Approach

**DOI:** 10.1155/2022/2598963

**Published:** 2022-10-26

**Authors:** Jhon Wilson A. Antido, Fresthel Monica M. Climacosa

**Affiliations:** Department of Medical Microbiology, College of Public Health, University of the Philippines Manila, Manila 1000, Philippines

## Abstract

*Streptomyces* species are considered to be the most prolific sources of various bioactive secondary metabolites that are important for antibiotic production. Here, we describe a modified integrated approach to isolate *Streptomyces* species from diverse soil habitats, such as dumpsite, garden, forest, grassland, and riverside in Calamba City, Laguna, Philippines. A total of 25 soil samples were collected from a depth of 0–20 cm using systematic random soil sampling. All soil samples were air-dried, crushed, pretreated with calcium carbonate, and incubated on a rotary shaker. Isolation of *Streptomyces* in soil samples was then performed using the standard serial dilution plate technique on starch casein agar supplemented with nystatin (50 *μ*g/ml) and ampicillin (5 *μ*g/ml). Identification of the *Streptomyces* isolates was done using a polyphasic method that includes morphological and biochemical characterization. A total of 103 morphologically and biochemically distinct *Streptomyces* were isolated from diverse soil habitats. The number of *Streptomyces* isolates varied in each collection site, with the highest number collected from dumpsite soil and the least from forest soil. Most of the hydrogen sulfide producers were noted to be isolated from dumpsite samples. Moreover, more *Streptomyces* were isolated in soil habitats at higher altitudes with a slightly acidic to alkaline pH and a temperature ranging from 29 to 33°C. Employing the modified integrated approach, we have isolated up to 10 times more *Streptomyces* compared to early studies. These *Streptomyces* isolates can be valuable for future drug discovery and development research.

## 1. Introduction

Microorganisms have long been a reputable source of secondary metabolites that have been successfully industrialized as medical drugs [1]. It is hypothesized that new metabolites can be discovered through intensive screening of the most prolific producers of chemically diverse secondary metabolites, such as actinomycetes [1–3]. Among actinomycetes, the majority of the novel antibiotics originated from different *Streptomyces* strains [4].

*Streptomyces* species are aerobic, filamentous, nonmotile, Gram-positive bacteria characterized by mycelial growth with more than 70% guanine-cytosine (GC) content in their DNA [5]. This genus comprises industrially and economically important microorganisms that can produce numerous novel bioactive secondary metabolites [6, 7]. Soil is the natural habitat of *Streptomyces* [6, 8], a physically, biologically, and nutritionally complex ecosystem often composed of complex carbohydrates [9]. *Streptomyces* species are widely distributed in soil environments [10]. However, studies highlighted the importance of collecting soil samples from unique and unexplored habitats for the isolation of possibly novel strains of *Streptomyces* [11–13]. More *Streptomyces* can also be isolated from nonagricultural soils [8, 14]. Similarly, environmental conditions of the soil habitats should be favorable to support the growth of *Streptomyces* [8].

In the Philippines, there is a wealth of evidence proving the existence of *Streptomyces* in soil habitats and the antibiotic-producing ability of the isolated strains. Several antibiotics have been discovered from the different *Streptomyces* strains isolated from the Philippine soil, such as erythromycin (ilotycin) [15], roseoflavin [16], and lagunamycin [17]. More recent local studies have focused on the isolation of antibiotic-producing *Streptomyces* from the marine environment [18, 19], while others have focused on the agricultural application of the compounds derived from different strains of *Streptomyces,* such as the promotion of plant growth [20] and crop protection [21]. Only a few local studies are directly involved in the isolation of *Streptomyces* from soil habitats [22, 23]. This study thus aims to investigate the effectiveness of a modified integrated approach for the isolation of *Streptomyces* from different soil habitats in a selected city (Calamba City, Laguna) in the Philippines.

## 2. Materials and Methods

### 2.1. Collection of Soil Samples

A total of 25 soil samples were collected from randomly selected diverse habitats (i.e., dumpsite, forest, garden, grassland, and riverside) in Calamba City, Laguna, Philippines. Systematic random soil sampling was used for the collection of soil samples, aided by grid maps generated using the ArcMap feature of ArcGIS 10.5 overlaid with maps from Google Maps for better visualization. Randomization of grid areas for dumpsite, forest, garden, and grassland was conducted using an online randomizer (https://www.randomizer.org/); samples were collected from the center of the randomized grids. Riverside samples were collected 1 m away from the river and 15 m away from each other. The environmental conditions of the collection sites were recorded in terms of geographic coordinates, altitude, soil temperature, and soil pH (Table 1). Five 400 g of soil samples were collected at each location from a depth of 0–20 cm. The samples were properly labeled and placed in tightly closed polyethylene bags to avoid spillage while being transported to the laboratory.

### 2.2. Pretreatment of Soil Samples

Soil samples were air-dried at room temperature for 7 days to remove moisture and reduce the population of bacteria other than *Streptomyces* species. Air-dried soil samples were then manually crushed and sieved through the sterilized mesh to get rid of large debris as previously described [24–26]. Soil-CaCO_3_ mixtures (10:1 w/w) were prepared and incubated for 2 days at 30°C in a closed sterile Petri dish as performed by Oskay [8]. Following the protocol of Njenga et al. [27], soil-CaCO_3_ mixtures (20 g) were suspended in 180 ml of sterile distilled water in a flask followed by incubation (28–30°C) on a rotary shaker at 200 rpm for 30 min.

### 2.3. Isolation of Pure Culture of *Streptomyces* Species

Isolation of *Streptomyces* species in soil samples was performed using the standard serial dilution plate technique on starch casein agar (SCA) (M2054, HiMedia Laboratories, India). In brief, all pretreated soil samples were serially diluted up to 10^−6^. To create a uniform suspension, each tube for serial dilution was vortexed. An aliquot of 1 ml of every dilution was plated out in triplicate and was overlaid with approximately 20–25 ml of SCA. After gentle rotation, isolation media were incubated at 28–30°C for 7 days. The culture media were prepared following the manufacturer's protocol and were supplemented with 50 *μ*g/ml of nystatin (Afunginal®, ACME Laboratories Ltd., Bangladesh) and 5 *μ*g/ml of ampicillin (Ampicin®, Sandoz International GmbH, Germany) to minimize the growth of contaminating fungi and bacteria, respectively. The total bacterial count (CFU/g of soil) and total *Streptomyces* count (CFU/g of soil) were also quantified following the procedure of Kizito and Nwankwo [14]. Purification of isolates was carried out by streaking colonies on new plates of SCA using sterile wire loops. The isolated pure cultures of *Streptomyces* species were transferred to the SCA slants and stored at 4°C until further use.

### 2.4. Characterization of *Streptomyces* Isolates

*Streptomyces* isolates were identified using a polyphasic method that includes cultural, morphological, and biochemical characteristics based on the guidelines recommended by Bergey's Manual of Systematic Bacteriology and the International *Streptomyces* Project [8, 24, 28]. All colonial morphological characteristics were observed on SCA and were used for classification and differentiation. The morphological characteristics consist of the color of aerial and substrate mycelia, melanoid pigment production of the colony, Gram stain appearance (i.e., viewed under 1000*x* magnification), and spore chain morphology. *Streptomyces* isolates were categorized into six color series (i.e., gray, white, red, yellow, green, and variable (brown, pink, orange, violet)), which were determined through comparison with the Inter-Society Color Council-National Bureau of Standards (ISCC-NBS) color charts [29]. Isolates were also either classified as melanoid producers (i.e., brown-black colonies) or melanoid nonproducers (i.e., colonies other than brown and black) [30]. Isolated strains were also grouped according to the shape of spore chains observed under light microcopy, such as rectiflexibiles (straight to flexuous), retinaculiaperti (open loops, hooks, or spirals consisting of one or two turns), and spirales (tight spirals) [24, 28]. Lastly, biochemical tests including catalase, motility, nitrate reduction, urea hydrolysis, hydrogen sulfide (H_2_S) production, indole production, methyl red, Voges-Proskauer, citrate utilization, and utilization of carbon sources (e.g., glucose, lactose, and sucrose) and gas production observed on triple sugar iron slant were conducted [6].

## 3. Results and Discussion

Table 1 shows the soil characteristics and the frequencies of bacterial and *Streptomyces* isolates from each soil habitat examined in this study. We observed that more *Streptomyces* species were isolated from soil habitats (e.g., dumpsite and garden) at higher altitudes, with slightly acidic to alkaline pH and temperatures ranging from 29–33°C, which is consistent with the reported optimum conditions that support *Streptomyces* growth [24, 30].

**Figure 1:**
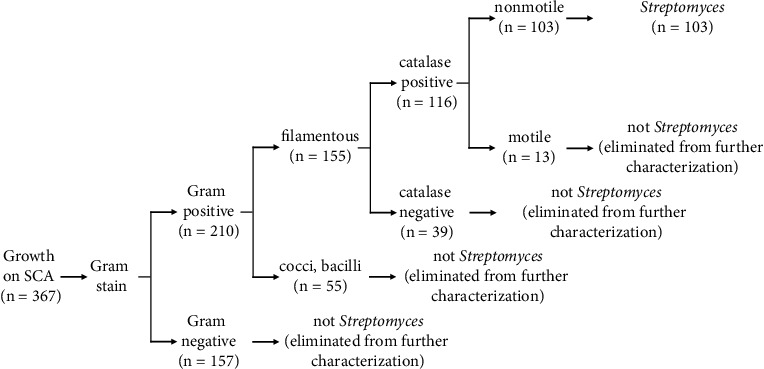
Polyphasic identification of *Streptomyces* isolates employed in this study (SCA: starch casein agar).

Bacterial load varied in each collection site, where the highest bacterial count of 4.6 × 10^7^ CFU/g of soil was from grassland, while the lowest count was from the forest with 6.2 × 10^6^ CFU/g of soil. Three hundred sixty-seven distinct colonial growths were seen on SCA post-incubation; 103 (28%) of these isolates were morphologically and biochemically distinct *Streptomyces* species (Figure 1), with the highest number isolated from dumpsite soil (35 out of 103). The same observation was noted in previous studies of Oskay [8] and Kizito and Nwankwo [14] where higher densities of *Streptomyces* have been isolated in nonagricultural soils like dumpsites.

**Figure 2:**
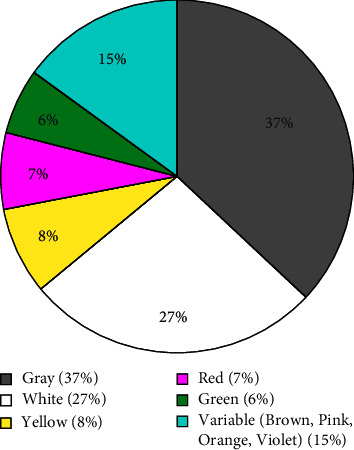
Percentage distribution of *Streptomyces* isolates according to colony color as observed on starch casein agar.

The color of the isolated *Streptomyces* colonies varied as depicted in Figure 2, although more than half are gray and white. Melanin production was seen on 14 *Streptomyces* colonies, notably from dumpsite, garden, and riverside soils. Barka et al. [30] suggest that melanin production has a vital role in enhancing *Streptomyces*' capability to adapt and survive in challenging and harsh environmental conditions.

**Figure 3:**
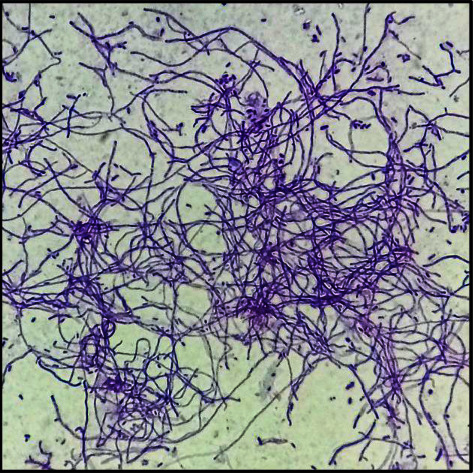
Representative photomicrograph of Gram stain of isolated *Streptomyces* (1000*x* magnification).

Microscopic examination revealed all 103 isolates are Gram-positive, filamentous with branching mycelia (Figure 3) and the majority possesses rectiflexibiles (74%) spore chains.  Table 2 displays biochemical test results consistent with the usual metabolic profile of *Streptomyces* species—catalase-positive, indole-negative, and can utilize glucose as their carbon source. Interestingly, most of the hydrogen sulfide producers (21 out of 37) came from the dumpsite, which is known to contain alternative sources of nutrients (e.g., food waste, paper, plastic) for microbial use with hydrogen sulfide as an end product [31, 32]. In addition, Long et al. [33] reported that the accumulation of hydrogen sulfide may cause an increase in soil pH, which favors the growth of *Streptomyces*. This could likewise explain the abundance of *Streptomyces* isolated from dumpsites as seen in this study.

Our approach to *Streptomyces* isolation combined pretreatment, enrichment, and selective inhibition methods. The pretreatment employed in this study consisted of desiccation, crushing, calcium carbonate supplementation, and incubation on a rotary shaker, as opposed to published studies that used these techniques singly or in combination [8, 24–27]. Starch casein agar as an enrichment culture medium [8, 34] and antibiotic supplementation (e.g., nystatin and ampicillin) for selective inhibition [25, 35] have been shown to be most effective in isolating *Streptomyces*. Performing this modified integrated approach, a substantially higher number of *Streptomyces* were isolated compared to similar studies (Table 3). Up to 10*X* more *Streptomyces* were isolated in this study compared to published literature using one pretreatment method alone and no antibiotic supplementation. Approximately 2–7*X* the number of *Streptomyces* isolates were isolated in our study employing the modified integrated approach, in contrast to early studies that applied antibiotic supplementation and one to two pretreatment methods only.

## 4. Conclusion

We have demonstrated in this study the utilization of a modified integrated approach for enhanced isolation of *Streptomyces* from soil habitats. We isolated 103 soil *Streptomyces* from different soil habitats in Calamba City, Laguna, Philippines, with the highest number collected from dumpsite soil. These isolates can be valuable for novel antibiotic discoveries. Further studies are now being conducted to investigate the potential antibiotic activity and to identify the isolates at the species level through molecular methods.

## Figures and Tables

**Table 1:** Location and environmental characteristics of the collection site with corresponding bacterial load from each soil sample.

Collection site	Geographic coordinates^*∗*^	Altitude level^*∗*^	Soil pH	Soil temperature (°C)	Total bacterial count (CFU/g of soil)	Total *Streptomyces* count (CFU/g of soil)	Number of *Streptomyces* isolates
Dumpsite	14°12′07″N 121°03′16″E	144 m	6.7	31.4	1.90 × 10^7^	7.0 × 10^6^	35
Riverside	14°11′59″N 121°03′07″E to 14°12′02″N 121°03′08″E	123.8 m	4.9	30.4	1.74 × 10^7^	4.6 × 10^6^	23
Garden	14°12′03″N 121°03′12″E	144 m	6.7	31.8	3.36 × 10^7^	4.0 × 10^6^	20
Grassland	14°12′11″N 121°03′20″E	139 m	5.8	32.4	4.60 × 10^7^	2.8 × 10^6^	14
Forest	14°12′41″N 121°07′45″E	41 m	7.1	32.6	6.20 × 10^6^	2.2 × 10^6^	11

^*∗*^Geographic coordinates and altitude were based on Google Maps.

**Table 2:** Biochemical characteristics of the *Streptomyces* isolates.

Biochemical tests	Results
Catalase	+
Motility	−
Urease	V (+: 62%/−: 38%)
H_2_S production	V (+: 36%/−: 64%)
Nitrate reduction	V (+: 92%/−: 8%)
Indole production	−
Methyl red	V (+: 31%/−: 69%)
Voges-Proskauer	V (+: 1%/−: 99%)
Citrate utilization	V (+: 82%/−: 18%)
Gas production	V (+: 13%/−: 87%)
Melanoid pigment production	V (+: 14%/−: 86%)
Utilization of carbon sources	
Glucose	+
Lactose	V (+: 69%/−: 31%)
Sucrose	V (+: 69%/−: 31%)

V: variable.

**Table 3:** The number of *Streptomyces* isolated in this study in comparison to previous studies.

Research studies	No. of soil samples	Pretreatment method/s	Culture media	Antifungal	Antibacterial	No. of isolated *Streptomyces*
THIS STUDY	25	4	SCA	✓	✓	103
[35]	8	1	SCA	—	—	10
[6]	—	1	SCA	—	—	10
[14]	25	1	CDA	✓	—	62
[36]	—	1	SCA	✓	—	79
[37]	—	1	GAA	✓	—	69
[38]	—	1	SCA	✓	✓	11
[39]	—	1	SCA	✓	✓	68
[40]	11	2	AIA	—	—	15
[41]	—	2	SCA	✓	✓	36
[27]	64	2	SCA	✓	✓	67

SCA: starch casein agar; CDA: Czapek-Dox agar; AIA: actinomycete isolation agar; GAA: glycerol asparagine agar.

## Data Availability

The data used to support the findings of this study are available from the corresponding author upon request.
